# Lessons Learned From Miami-Dade County's COVID-19 Epidemic: Making Surveillance Data Accessible for Policy Makers

**DOI:** 10.1097/PHH.0000000000001364

**Published:** 2021-03-10

**Authors:** Roy Williams, Zoran Bursac, Mary Jo Trepka, Gabriel J. Odom

**Affiliations:** University of Florida, Gainesville, Florida (Dr Williams); Department of Biostatistics (Mr Williams and Drs Bursac and Odom), Research Center for Minority Institutions (Drs Bursac and Trepka), and Department of Epidemiology (Dr Trepka), Robert Stempel College of Public Health & Social Work, Florida International University, Miami, Florida; and Department of Public Health Sciences, Leonard M. Miller School of Medicine, The University of Miami, Miami, Florida (Dr Odom).

**Keywords:** COVID-19, public health surveillance, public policy, severe acute respiratory syndrome coronavirus 2, surveillance

## Abstract

COVID-19 represents an unprecedented challenge to policy makers as well as those entrusted with capturing, monitoring, and analyzing COVID-19 data. Effective public policy is data-informed policy. This requires a liaison between public health scientists and public officials.

**Objective:**

This article details the experience, challenges, and lessons learned advising public officials in a large metropolitan area from March to October 2020.

**Methods:**

To effectively do this, an R Markdown report was created to iteratively monitor the number of COVID-19 tests performed, positive tests obtained, COVID-19 hospitalization census, intensive care unit census, the number of patients with COVID-19 on ventilators, and the number of deaths due to COVID-19.

**Results:**

These reports were presented and discussed at meetings with policy makers to further comprehension.

**Discussion:**

To facilitate the fullest understanding by both the general public and policy makers alike, we advocate for greater centralization of public health surveillance data, objective operational definitions of metrics, and greater interagency communication to best guide and inform policy makers. Through consistent data reporting methods, parsimonious and consistent analytic methods, a clear line of communication with policy makers, transparency, and the ability to navigate unforeseen externalities such as “data dumps” and reporting delays, scientists can use information to best support policy makers in times of crises.

## Introduction and Purpose

**FIGURE 1:**
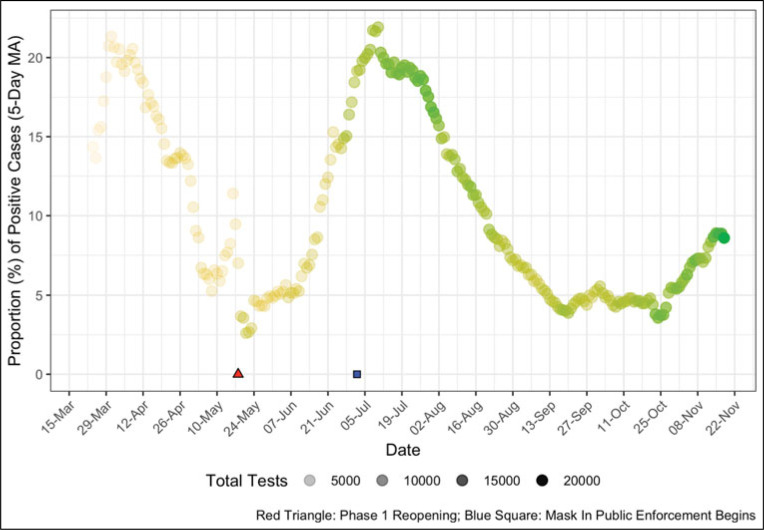
Five-Day Moving Average of Proportion of All COVID-19 Tests That Were Positive and Number of Daily Tests (Depicted by the Color and Opacity of Data Points) by Day From March 20 to November 18, 2020 Abbreviation: MA, moving average.

During fall 2019, a novel coronavirus, later named SARS-Cov-2, emerged in Wuhan, China. The first reported case of COVID-19, the disease caused by this coronavirus, was reported in the United States on January 20, 2020.^1^ By the end of that month, cases had already been recognized in 19 countries.^2^ By March 26, there were 85 356 reported cases of COVID-19 in the United States^3^ and 178 reported cases in Miami-Dade County. In response to this growing number of COVID-19 cases, the Mayor of Miami-Dade County (population 2.72 million^4^) issued a “Safer at Home” order on March 26,^5^ and the Governor of Florida issued a similar proclamation on April 1.^6^ These and associated orders led to shutting down of nonessential businesses and schools. During the shutdown, the percentage of positive COVID-19 test results among tested people in the county declined significantly, as did the number of positive tests (see Figure 1).

On March 16, before the “Safer at Home” order, the Florida Department of Health (FLDoH) published the very first daily COVID-19 report for the state (including counts for each county)^7^ and released a dashboard of daily positive and negative test results shortly thereafter.^8^ The timeliness of these reports was unusual for Florida public health surveillance; previously, the most frequently issued communicable disease surveillance reports in Florida (eg, influenza surveillance reports) were issued weekly.^9^ These actions to collect, manage, and disseminate timely and accurate surveillance data were critical to inform public health action.^10^ However, despite the widespread availability of these data, it soon became clear that the public, media, and many policy makers had difficulty understanding these data. During the first week of May, the City of Miami Division of Emergency Management reached out to the authors for assistance and expertise; our team members include a medical epidemiologist, a senior consulting biostatistician, a data scientist, and a biostatistics graduate student. In particular, Miami's elected and appointed officials wanted help in understanding trends in the COVID-19 testing and hospitalization data.

In response to the needs articulated by the city of Miami, the Miami COVID-19 Project^11^ began (http://www.miamicovidproject.com).* This project involved analyzing and reporting trends in testing data and positivity rates, hospitalization data (including intensive care unit [ICU] and ventilator use), and deaths. These reports, which were updated 2 to 3 times a week from May until November and subsequently once a week, became a key source of information used by policy makers from the cities of Miami, Miami Beach, Miami Gardens, the Miami-Dade League of Cities, and other municipalities in the county. The authors of this report have met with leaders of the aforementioned cities 1 to 3 times a week since the spring of 2020 to deliberate about strengthening and relaxing various control measures. In this article, we present a few questions raised by local elected and appointed officials and the reports and figures that we presented to help us answer those questions. The purpose of this article is to describe how surveillance data were made accessible to policy makers in one large urban area and the lessons learned during this process.

## Data and Methods

### Primary data source I, FLDoH reports

The number of laboratory tests performed, the number of positive and negative tests obtained, the percentage of positive tests, and the number of reported deaths per day were obtained from the daily FLDoH's county reports.^12^

### Primary data source II, Florida's Agency for Health Care Administration Hospital Census

Information regarding daily COVID-19 hospitalizations, COVID-19 ICU census, COVID-19 ventilator use, as well as daily COVID-19 discharges and admissions were provided via the Florida's Agency for Health Care Administration's (AHCA's) Emergency Status System (ESS).^13^

### Development of a report

An R Markdown^14^ report (available at the authors' technical development Web site: https://github.com/Rwilli5/MiamiCovidProject) was created to iteratively report trends in COVID-19 number of tests, positive tests, positivity rate, hospitalizations, ICU census, ventilator use, and deaths. The scripts were written in R,^15^ and the report was configured to automatically print updated date metrics and slopes.

### Measures

#### Testing

Overall testing was defined as the number of positive and negative COVID-19 PCR (polymerase chain reaction) and antigen laboratory test results the FLDoH received per day. The percent positive is the number of people who test positive for the first time on a given day excluding people who have previously tested positive, divided by all the people tested that day.^12^ Only Florida residents were included.

#### Hospitalizations

AHCA's ESS includes all COVID-19 hospitalizations of both residents and nonresidents hospitalized at class I-IV hospitals, inclusive of long-term care facilities and rehabilitation centers, excluding those in the Veteran Administration's hospital system.

#### Deaths

The count of COVID-19 deaths was obtained daily from the FLDoH's state line list of Florida residents.^16^

### Data science methods

The Tidyverse suite^17^ of R packages was used to import and process data from both AHCA ESS reports and FLDOH reports into usable data tables. The data were formatted by county and date. Five-day moving averages were calculated starting on the first day to smooth the data and provide more robust trend information.^18^ Because we were updating and presenting these reports 3 times per week from May until November 2020, many decision makers were confused by the day-to-day variability in counts and proportions and, in some instances, placed too much decision-making weight on small perturbations in the daily data. Five-day moving averages allowed us to balance sensitivity with respect to short-term changes in viral spread while still reducing the influence of daily values on policy decisions. Linear and polynomial models were fitted to case counts and percent positivity for the most recent 2 weeks of data as needed to describe recent trajectories for those measures (figures of 14-day data window included on the authors' Web site). In addition, locally estimated scatterplot smoothing^19^ (LOESS) curves and generalized additive model^20^ (GAM) curves were applied to highlight macro-level data trajectories and describe the outcome behavior over longer periods of time.

## Results

In this section, we discuss 3 broad groups of questions raised by local officials during 2020: testing, hospitalizations, and deaths. In May and the first part of June, local officials were concerned with adhering to guidelines from the White House concerning phase I reopening of the economy. Of paramount importance to reopening were questions of the 14-day “trajectory”^21^ of the county epidemic. Starting in mid-May, the county updated a dashboard of testing and hospitalization data daily,^22^ but this report did not include rates of increase or decrease, offered very little discussion to guide policy decisions, and (as mentioned later) contained errors. Because policy decisions were being made primarily based on this 14-day data window, the following testing data were prepared for discussion 1 to 3 times per week, depending on the frequency requested by local policy makers: (1) average daily change in the number of COVID-19 tests reported over 2 weeks; (2) average daily change in the number of positive COVID-19 tests reported over 2 weeks as well as weekly the average number of positive COVID-19 test results; and (3) average change in the percentage of positive test results as well as the weekly average percentage of positive test results. Average daily changes were calculated and reported to understand what the slopes of curves were, that is, whether or not the rate of increase or decrease was speeding up or slowing down. Every 2 to 3 days, we updated the data and presented figures for the most recent 14 days, explaining to local officials how to interpret these data and offering data-driven critiques of proposed policy changes. All data and figures used are publicly available at http://www.miamicovidproject.com.

In addition to questions concerning 2-week pandemic trends surveillance data, figures for the entire outbreak were presented to allow for comparison of the current week with historical trends. The data formats reported here are examples of how data were reported to local policy makers.

### Positive cases and tests

During many of our conversations with policy makers, we were asked how the current state of the pandemic spread compared with the “first wave” (which triggered the state “Safer at Home” order). To answer this question, we would present a version of Figure 1 each week, which depicts the proportion of positive COVID-19 test results of all COVID-19 tests throughout the pandemic outbreak in Miami-Dade County. The color and opacity of the dots represent the total number of daily tests completed: days with lower total test counts (<5000) are shown in sheer yellow, while days with higher total test counts (>15 000) are shown in bold green. The red triangle on May 18, 2020, represents the first phase 1 reopening for Miami-Dade County (the first step to lift the “Safer at Home” lockdown). In addition, policy makers considered the unbridled June pandemic spread shown in this figure as sufficient evidence to begin civil enforcement of a “Mask in Public” rule on July 2, 2020 (blue square),^23^ a critical intervention necessary to stem the rampant viral spread. While the trajectory and height of the positivity rate during second spike (June-August) mirrored that of the first spike (March-April), the total community testing numbers during the second spike were up to 8 times greater than during the first spike (eg, 2074 total tests on March 31 compared with 16 903 total tests on July 31).

Several problems were identified using the testing data. First, because of a previously inaccessible data dictionary, county officials were initially incorrectly calculating test positivity rate. Consequently, they erroneously reported a peak positivity rate of 33.5% on July 9, with a 14-day average of 26.6% on July 14,^24^ which was sensationalized in the national media.^25^ The positivity rate for July 9 was actually 20.2% (2349 positive tests out of 11 612 total shown in the July 14 FLDoH report)^26^ and the 14-day average for July 14 was 20.3% (34 300 positive tests out of 168 734 total, as shown on our Web site). The discrepancy between county-generated and FLDoH-generated rates led to confusion among local policy makers. In addition, there is a large percentage of missing observations for race/ethnicity of cases. As of this writing, 37% of cumulative COVID-19 cases in Miami-Dade County had “other” or “unknown” race and 20% had unknown ethnicity.^27^ The incomplete data on race/ethnicity for cases make it difficult to monitor COVID risk by race/ethnicity. This is particularly important because non-Hispanic Blacks and Hispanics have been disproportionately affected by COVID-19 incidence and mortality.^28^,^29^

### Daily hospitalizations

**FIGURE 2:**
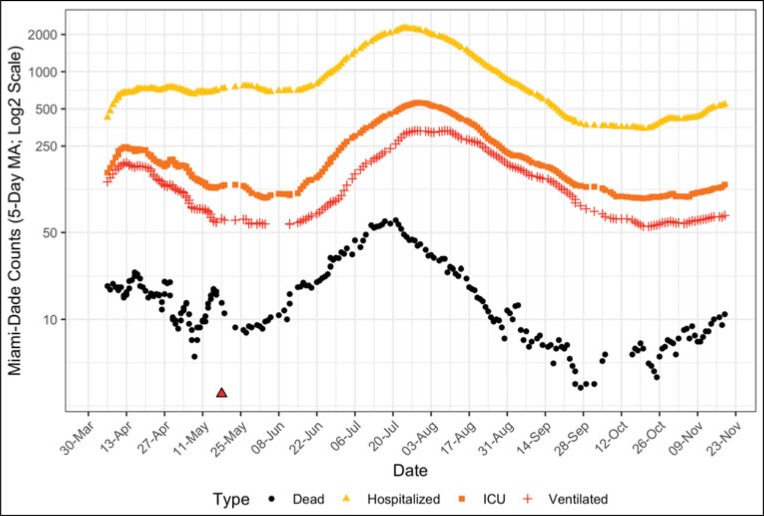
Daily Hospital Census, ICU Occupancy, Ventilator Use, and Deaths Attributed to COVID-19 Infection From April 2 to November 19, 2020 Abbreviations: ICU, intensive care unit; MA, moving average.

As this pandemic progressed into its second wave in June and July, officials shared serious concerns about potential shortages of ICU beds and ventilators. They asked us to explore trends over time in total daily hospital resource use by those COVID-19 positive. We prepared figures showing long-term trends in hospitalizations, ICU bed use, and ventilator use. Figure 2 shows the logarithmically transformed 5-day moving average of counts of persons positive for COVID-19 infection who were hospitalized (yellow), in intensive care (orange), and on ventilators (red). The log transformation was necessary to show all 3 measures on the same graphs, but the vertical axis labels show the raw counts. Of note, while the second spike in cases (as measured in the proportion of positive tests in the community) was in early July, the peaks in hospitalization census, especially among the most critical patients, occurred almost a month later. While we include daily counts of deaths in this figure for publication, we did not have access to full mortality data at the height of the second wave (as explained later). Thus, hospitalization data were critically important to local decision makers as a key indicator of disease burden. A continuing problem was intermittent lack of access to ESS data, particularly on weekends. In addition, a data dictionary did not accompany ESS data, requiring reliance on expert opinion from local emergency service officers to confirm that we are interpreting AHCA ESS data features correctly.

### Deaths

Although deaths are a lagging indicator of disease activity, local policy leaders were acutely interested in understanding the level of mortality so that they could accurately communicate the severity of the outbreak. These officials repeatedly questioned how the number of cases and hospitalizations could rise so sharply in June but daily deaths for the same time frame *appeared* to be decreasing—to the point that national and local opinion pieces were written to explain how cases could be on the rise while deaths were “falling.”^30^,^31^ Local officials expressed their concerns that the general public would see the number of deaths declining while the number of cases increased and erroneously assume that the virus had become less deadly.

**FIGURE 3:**
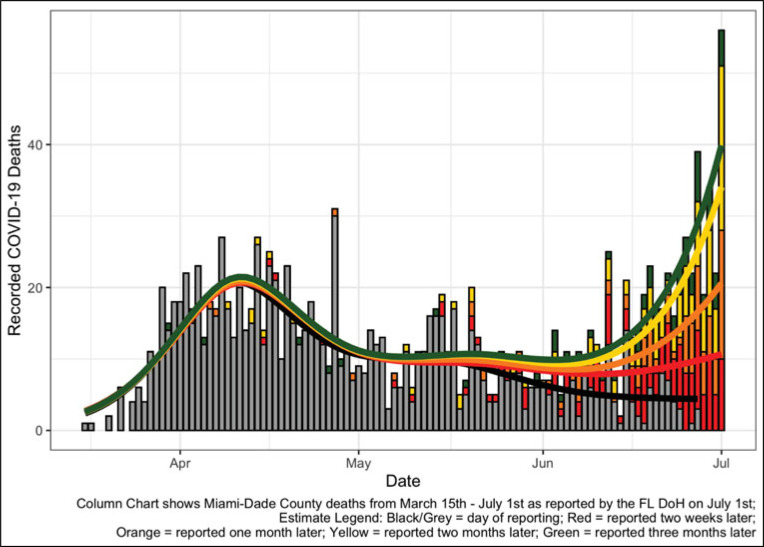
Number of COVID-19 Deaths by Date of Death as Reported on July 1 (Black), 2 Weeks Later (Red), 1 Month Later (Orange), 2 Months Later (Yellow), and 3 Months Later (Green); Miami-Dade County, March 1-July 1, 2020 Abbreviation: FLDoH, Florida Department of Health.

With the aid of our local FLDoH office, we investigated the counts of reported deaths. Because of the large number of deaths, reporting of COVID-19 deaths was delayed by more than 6 weeks (see Figure 3). While the FLDoH dashboard reported numbers of deaths by date of death, we truncated the figures on the number of deaths in our report because delays in classification of deaths gave the incorrect impression that deaths were *declining* throughout July and August, when they were in reality increasing (see Figure 3). Note that ascribing an underlying cause of death is difficult and is sometimes imprecise,^32^ and the mortality data that we had available to us were only counts. This allowed us to follow trends, but it was not possible for us to assess the level of misclassification of cause of death or changes in the likelihood of misclassification throughout the course of the pandemic.

Figure 3 shows the number of reported deaths compared with actual deaths between March 1 and July 1 for Miami-Dade County. The black bar chart (*black line*) depicts the count by day (*smoothed average*) of deaths over the course of the pandemic as reported on July 1. The green bar chart (*green line*) shows the count by day (*smoothed average*) of deaths over the course of the pandemic as reported on October 1; that is, if we had a time machine and traveled from July 1 to October 1, we would have seen the green bar chart as the death tally for the time period shown on the graph. We used this figure to explain to policy makers and members of the news media that death counts were not, in fact, going down during the height of the second wave and immediate aftermath.

## Discussion and Lessons Learned

There are a number of challenges, strategies, and lessons that can be derived from informing policy makers during an unprecedented public health crisis such as the COVID-19 pandemic. These challenges and lessons fall into 2 broad categories, concerning (1) centralized and transparent data sources and (2) informing people of data limitations.

### Need for a centralized and transparent data repository

Foremost, timely and accurate communication of surveillance data requires parsimony, making critical information accessible without burdening the recipient with unnecessary supplemental information. Given the scale and severity of this pandemic in the United States and worldwide, there was an enormous push for data collection and reporting at every level. However, during this pandemic, differences in how data were reported created confusion. For example, differences in reported hospitalizations between the AHCA and county-collected data led to uncertainty about how many ICU beds were actually available. A more complete solution would be greater centralization of data collection and storage (eg, one source of surveillance data and daily hospitalization census data) to avoid redundancy, discrepancy, and streamline information to those with a need to know.

Another key principle to cultivate public trust and credibility is that of data timeliness and transparency. In situations such as COVID-19 where misinformation is often pervasive, transparency is essential to gaining public trust. Open-source software and the free distribution of nonsensitive data can aid in ensuring public trust, public understanding, and public policy compliance. In contrast, a lack of data, data dictionaries, and data source/analysis transparency can fuel misinformation and confusion among public officials and the general public—as we observed in July with respect to case rates. Data sources and classification methods need to be clarified to the fullest extent possible to prevent the spread of misinformation and foster a culture of transparency and an informed public.

Throughout the pandemic, a major challenge for surveillance data users was lack of documentation about how FLDoH was calculating the proportion of positive test results. More detailed documentation in reports would assist data users in data interpretation. Furthermore, timely and accurate information is paramount to support data-driven decision-making. This requires central agencies to be transparent in their changes in either analytic methodology or surveillance methodology when creating reports so that public health experts can stay abreast of any changes in reporting criteria or data format.

### Limitations of reported data

Having freely accessible data is not enough by itself. The “raw” data are exactly that: raw, unprocessed, and not conducive for consumption by the general public. Even with a consistent reporting methodology, the real counts observed can change from day to day in often bizarre ways, simply by random chance. Despite using consistent case definitions and analytic methods to minimize internal challenges to data informed policy, there are inevitably external pitfalls.

One of the biggest challenges has been “data dumps.” Data dumps can loosely be defined as an often untimely release of large amounts of historic data that were previously missed or not reported (often from technical system issues or a laboratory not reporting for an extended period of time).^33^,^34^ Data dumps can threaten the integrity of an analysis by artificially altering the number of cases and other metrics for certain days. Observing an unusually large number of overall tests, or positivity rate that is not in line with long-term trends, can be a good “red flag” indicator of a dump. If a data dump has truly occurred, every attempt needs to be made to back-allocate counts to their appropriate days. When this is not possible, data smoothers—such as moving averages, in conjunction with regression smoothers—such as LOESS or GAM models, can be used to displace the effect of a data dump over a range of dates. Smoothing reduces the variability in data and allows for a more robust picture of a given trend.

Moreover, when presenting data analysis results to public officials, great care is needed in explaining both the analysis and its limitations. Similar to the challenges presented by data dumps, delays in reporting of deaths can render a disconnection between what data purport to show and reality. For example, consider the death data in Miami-Dade County: by mid-August, there was a delay in the reporting of deaths by more than 6 weeks. If policy makers were looking at a graph of deaths at this time, they would erroneously conclude that deaths had fallen dramatically when, in fact, deaths were rising significantly. In Florida, the sheer number of deaths requiring certification delayed the reporting process by more than 6 weeks (see Figure 3). A solution to this is to discern how long the delay is and how it may change over time and to present data that are reasonably accurate up until a certain point. While this may not be a perfect solution, it is a better alternative than using incomplete data that can be misleading. Scientists advising officials need to take great care in examining the data presented and look for delays or other externalities.

In summary, we believe that public health scientists should use their experience to best support policy makers in times of crises. This support will be effective through consistent data reporting methods, parsimonious and consistent analytic methods, a clear line of communication with policy makers, transparency, and the experience necessary to navigate unforeseen externalities such as data dumps and reporting delays. However, for this to occur, there needs to be both data availability at the highest level and public officials who understand the value of data-informed policy. In the future, based on our experiences, we recommend greater centralization of data, objective operational definitions of metrics, and greater interagency communication to best guide and inform policy makers.

**Implications for Policy & Practice**

We call for state and local government agencies to:

- *Increase centralization of public health surveillance data.* At the time of publication, public health surveillance data concerning the COVID-19 pandemic are hosted by at least 3 different state and local agencies in the state of Florida and counties of Miami-Dade, Broward, and Palm Beach (the Greater Miami metropolitan area). *Any data not including protected health information should be easily accessible to the general public from a central data repository in a standard usable form*.
- *Define operational metrics clearly and objectively, and publish them for open public review*. Over the course of this pandemic, we have had serious challenges understanding the definitions of certain critical reported metrics. In addition, in the state of Florida particularly, some debate has arisen over the definitions of what exactly constitutes a COVID-19 case or death. *Data need explicit dictionaries containing ethical and scientifically rigorous justification in addition to robust and informative examples*.
- *Facilitate greater inter- and extra-agency communication to best guide and inform policy makers*. During the initial lockdowns, many elected officials we spoke with expressed frustration and ignorance of the entire situation. Their questions remained unanswered. *One single state agency or department should be responsible to triage questions and disseminate consistent information during public health emergencies*.

## Footnotes

* Data sources and code availability: The authors' Web site shows figures and data, updated weekly: http://www.miamicovidproject.com. The code necessary to build this Web site is available at https://github.com/Rwilli5/MiamiCovidProject. The authors provide supplemental data sets and code to build figures at https://github.com/gabrielodom/miami_COVID19_case_study.
